# Absence of proximal muscle weakness, dysarthria, and facial diplegia suggests Guillain–Barre syndrome rather than CIDP

**DOI:** 10.1186/s41983-022-00598-z

**Published:** 2022-12-15

**Authors:** Josef Finsterer

**Affiliations:** Neurology and Neurophysiology Center, Postfach 20, 1180 Vienna, Austria

**Keywords:** SARS-CoV-2, COVID-19, Vaccination, CIDP, Guillain–Barre syndrome

## Abstract

The aim of this letter to the editor is to discuss the etiology and pathophysiology of chronic inflammatory demyelinating polyneuropathy (CIDP) in two patients, of whom one experienced a mildly symptomatic SARS-CoV-2 infection 2 months prior to onset of the CIDP (patient-1), whereas patient-2 developed CIDP with bilateral facial palsy 19 days after receiving a shot of an RNA-based anti-SARS-CoV-2 vaccine. Causality between the SARS-CoV-2 infection and CIDP in patient-1 remains unsupported and the diagnosis CIDP in patient-2 remains questionable. Although delineation between CIDP and GBS is not clear cut, bilateral facial palsy and absence of proximal involvement suggest GBS rather than CIDP.

## Introduction

With interest we read the article by Fotiadou and colleagues about two patients with chronic inflammatory demyelinating polyneuropathy (CIDP) after an infection with SARS-CoV-2 2 months earlier (patient-1) and 19 days after vaccination with an mRNA-based anti-SARS-CoV-2 vaccine (Ad26.COV2.S) [1]. The study is appealing but raises concerns and comments.

## Main text

We disagree with the diagnosis CIDP in patient-2 [1]. The patient presented with dysarthria, facial diplegia, foot extensor weakness, and facial and acral paresthesias [1]. The first argument against CIDP is that the patient had facial diplegia and bilateral prosoplegia [1]. Facial diplegia has been only rarely reported in patients with CIDP. Therefore, facial diplegia rather suggests that the patient had Guillain–Barre syndrome (GBS) with cranial nerve involvement than CIDP. In general, cranial nerve involvement in typical CIDP is rather the exception than the rule [2]. Only in nodopathies involvement of the cranial nerves is a common feature [2]. A second argument against CIDP is that the disease course was not progressive during 8 weeks as requested by the EFNS criteria for CIDP [1]. The authors mention an “initial episode” of undescribed duration and that symptoms “recurred” 58 days after the initial episode, suggesting that there was no continuous progression during 8 weeks. A third argument against CIDP in patient-2 is that there was no proximal muscle weakness [2]. Patient-2 has been described with weakness only of foot extensors [1]. No other muscles developed weakness during the disease course. Diagnosing typical CIDP, including acute-onset CIDP (A-CIDP), requires presence of proximal and distal muscle weakness [2]. A-CIDP is less likely to have autonomic nervous system involvement, facial weakness, a preceding infectious illness, or the need for mechanical ventilation, in comparison with AIDP [3].

We also disagree with the description in Table 2 that involvement of respiratory muscles is absent in CIDP [1]. Although more rarely reported in CIDP than in GBS, there is involvement of respiratory muscles in CIDP and some patients may even require ventilatory support [4].

We should be informed if dysarthria was due to facial diplegia, involvement of cranial nerves IX and X, or if dysarthria was due to involvement of the brainstem. A subtype of GBS manifests with bulbar involvement (Bickerstaff brainstem encephalitis (BBE) [5]. Missing in this respect is cranial magnetic resonance imaging (MRI) with contrast medium to eventually demonstrate thickening or enhancement of cranial nerve roots.

Furthermore, we disagree with the notion that CIDP in patient-1 was due to an infection with SARS-CoV-2 [1]. Latency between onset of COVID-19 and onset of CIDP was 2 months, a time span too long to justify considerations about a causal link. The long latency between COVID-19 and onset of CIDP rather suggests that CIDP was due to causes other than SARS-CoV-2.

Missing in patient-1 is the determination of cytokines, chemokines, and glial fibrillary acidic protein (GFAP) in patient-1. These CSF parameters have been reported elevated in GBS due to an infection with SARS-CoV-2 [6].

Missing are also the results of contactin and neurofascin antibody titers to assess if the two index cases had a nodopathy rather than a SARS-CoV-2 infection/vaccination-related disease.

## Conclusions

Overall, the elegant study has several limitations that challenge the results and their interpretation. These limitations should be addressed to broaden the conclusions. Delineation between CIDP and GBS is challenging. Involvement of cranial nerves rather suggests GBS than CIDP.

## Data Availability

All data reported are available from the corresponding author.

## Abbreviations

BBE: Bickerstaff brainstem encephalitis
CIDP: Chronic inflammatory demyelinating polyneuropathy
CSF: Cerebrospinal fluid
EFNS: European Federation of neurological sciences
GBS: Guillain–Barre syndrome
GFAP: Glial fibrillary acidic protein
MRI: Magnetic resonance imaging
